# 非小细胞肺癌患者的肿瘤突变负荷异质性研究进展

**DOI:** 10.3779/j.issn.1009-3419.2021.102.12

**Published:** 2021-04-20

**Authors:** 米热阿依·阿布都热孜克, 鹏 陈

**Affiliations:** 300060 天津，天津医科大学肿瘤医院肺部肿瘤内科，国家肿瘤临床医学研究中心，天津市肿瘤防治重点实验室，天津市恶性肿瘤临床医学研究中心 Department of Thoracic Oncology, Tianjin Medical University Cancer Institute and Hospital, National Clinical Research Center for Cancer, Tianjin Key Laboratory of Cancer Prevention and Therapy, Tianjin Clinical Research Center for Cancer, Tianjin 300060, China

**Keywords:** 肺肿瘤, 肿瘤突变负荷, 异质性, Lung neoplasms, Tumor mutation burden, Heterogeneity

## Abstract

抗程序性死亡蛋白配体-1(programmed death ligand 1, PD-L1)是靶向免疫治疗的著名生物标志物，然而不同肿瘤PD-L1的表达与治疗疗效的关系不同，部分PD-L1阴性患者接受免疫治疗有效，而部分PD-L1阳性甚至强阳性的非小细胞肺癌(non-small cell lung cancer, NSCLC)患者并不能从免疫治疗获益。通过全外显子测序(whole exome sequencing, WES)的肿瘤突变负荷(tumor mutation burden, TMB)是一种近期获得美国食品药品管理局批准的免疫治疗生物标志物。本文现对影响NSCLC患者的TMB的因素进行简单的综述，从而有助于临床医师根据TMB值筛选出免疫治疗的真正优势人群。

## Abstract (en)

Programmed death ligand 1 (PD-L1) is a well known biomarker for targeted immunotherapy. However, the relationship between the expression of PD-L1 and the immunotherapy efficacy is not always consistent in different cases. Some patients who are PD-L1 negative still can benefit from immunosuppressive therapy, while some non-small cell lung cancer (NSCLC) patients with PD-L1 positive, even strongly positive, can not. Therefore, PD-L1 is not a completely reliable immunotherapy biomarker. Tumor mutation burden (TMB) estimated by whole exome sequencing (WES) is a biomarker recently approved by Food and Drug Administration (FDA). In this paper, we briefly reviewed the factors that result in the variaty of TMB in order to improve the reliability of the TMB and help clinicians to select patients who can get benefit from immunotherapy more wisely.

随着免疫治疗时代的到来，免疫治疗的生物标志物正在逐步成为当前研究的热点。肿瘤突变负荷(tumor mutation burden, TMB)，一种新兴的免疫治疗标志物，被定义为肿瘤细胞基因组中某一特定区域中的体细胞突变总数，精确定义随测序区域的大小以及突变性质的不同而变化^[1-3]^。理论上，肿瘤细胞中的的体细胞突变越多，TMB值就会越高，形成新抗原的可能性也越大。但是并非细胞表面的所有新肽都具有免疫原性，因为体细胞突变后产生的肽链中只有一小部分会经加工后加载到主要组织相容性复合体(major histocompatibility complex, MHC)复合物上，其中只有部分能够被T细胞识别，引起抗肿瘤免疫反应^[4, 5]^。第二代测序(next-generation sequencing, NGS)对TMB的评估涉及全基因组测序(whole genome sequencing, WGS)、全外显子测序(whole exome sequencing, WES)或者靶向基因面板。TMB在临床实践中更可能由基于不同面板的NGS评估^[6, 7]^。目前已被美国食品药品监督管理局(Food and Drug Administration, FDA)批准用于TMB检测的面板包括FoundationOne面板和MSK-IMPAKT面板^[8, 9]^。WES覆盖了编码区大概2.2万个基因(约18万个外显子，基因组大小为30 Mb)，占整个基因组的1%^[10]^。由于WES成本较高、样本需求量较大、数据分析较复杂，在临床应用中受限^[11]^。因此，采用聚焦于大量肿瘤相关基因的靶向测序面板进行肿瘤基因组分析在临床检测中更加常见。与WES相比，靶向面板可以达到更深入的测序。也有越来越的研究^[12, 13]^证明基于靶向面板的TMB(targeted panel sequencing-based TMB, pTMB)与基于WES的TMB(WES sequencing-based TMB, wTMB)高度相关。但是，不同面板之间TMB报告缺乏协调性，分析前因素也会对TMB的评估产生重大影响，例如样本的收集和处理、样本的质量和纯度、福尔马林固定石蜡包埋组织样本(formalin-fixed, paraffin-embedded samples, FFPE)诱导的脱氨伪影以及DNA文库的制备等^[13-15]^，以上均不可避免的导致了TMB值的异质性。为了筛选出真正能够从免疫治疗中获益的人群，区分出可靠的TMB值，临床医生需要对导致TMB异质性的因素有进一步的认识。

## 1 不同实验室及平台间TMB的异质性

NGS检测TMB的精确度及特异度取决于肿瘤内的突变频率及所使用的面板能够实现的测序深度及覆盖范围。2019年欧洲肿瘤内科学会(European Society for Medical Oncology, ESMO)推荐精确评估TMB面板所需最小覆盖深度为 > 200倍^[16]^。目前使用最广泛的靶向面板包括含有468个目标基因的MSK-IMPACT面板(基因组大小为1.22 Mb)以及含有324个目标基因的the Foundation Medicine面板(基因组大小为1.2 Mb)。Rizvi及其合作者的研究^[17]^发现，使用MSK-IMPACT面板时，肿瘤样本的平均测序覆盖率达744倍，使用WES时，覆盖率为232倍。由此可以得出，在肿瘤细胞纯度较低或异质性较高的样品中，相比WES，进行靶向面板可以实现更深入的测序以保持较高灵敏度^[16]^。Jan Budczies等^[18]^ 在2020年通过筛选已发表的包含NSCLC患者检测结果在内的实验室基于pTMB、建立误差模型以及量化影响pTMB结果的各个混杂因素，提出与面板大小相关的随机误差是最重要的误差来源，面板尺寸每增加一倍，随机误差将减少1.4倍，减半随机误差需要将面板大小增加4倍。11个具有不同NGS目标基因面板和生物信息学方法的实验室参加了癌症研究之友(Friends)TMB协调项目的第一阶段，该项目初步分析了包含肺腺癌在内的8种癌症类型(8种癌症的TMB值的动态范围均在40 mut/MB以内)，通过对这些癌症进行单独评估，Firends提出在肺腺癌中，pTMB与wTMB无明显差异^[19]^。2019年，Endris等^[20]^提出对于TMB遵循双峰分布的肿瘤，小于1 Mbp的测序面板足以可靠地区分出TMB高与TMB低的人群，然而由于绝大多数NSCLC患者的TMB值呈连续分布，对于这种情况，需要较高分析能力的面板来更靠地区分TMB高和TMB低的人群。2019年，Buchhalte等^[21]^通过建立模拟面板，比较了0.5 MBP、1 MBP和1.5 MBP大小的面板对TMB检测的敏感性和特异性。在此研究中，对于肺腺癌，用三种不同大小的面板检测TMB的敏感性分别为0.78、0.82和0.85，特异性分别为0.89、0.93和0.94。

## 2 肿瘤异质性对TMB影响

2017年，Jamal-Hanjani等^[22]^对100例在全身治疗前切除的早期NSCLC肿瘤进行了327个区域全外显子组测序，指出体细胞拷贝数改变和突变存在广泛的肿瘤内异质性。因此，TMB也避免不了会受到肿瘤异质性的影响。2018年，Jia等^[23]^通过对15例NSCLC患者的肿瘤组织的多个区域进行分析，发现基因突变的数量在同一患者的不同标本上有很大的差异，提出肿瘤局部突变负荷不能有效地预测肿瘤的整体免疫激活状态。在晚期NSCLC患者中，在原发肿瘤和转移瘤之间也存在着不可忽视的突变异质性^[24]^。Kazdal等^[25]^在2019年通过24例肺腺癌的多区域样本和5例患者的匹配原发肿瘤、局部淋巴结转移和远处转移的样本分析TMB(使用TruSight Oncology 500 targeted sequencing panel)的空间异质性，发现在30%的患者中TMB有明显的瘤内异质性(最大差异可达14.13 mut/Mbp)，淋巴结源性TMB评分显著降低，原发肿瘤与远处转移瘤之间存在TMB差异，但差异不显著。2018年Gandara等^[26]^首次证实了从血液中测得的TMB(blood-based tumor mutational burden, bTMB)是接受阿替唑单抗治疗的NSCLC患者无进展生存期(progression-free survival, PFS)的预测性生物标志物，并指出外周血中循环肿瘤DNA(circulating tumor DNA, ctDNA)中必须包含等位基因突变频率≥1%时bTMB才有意义。2019年Wang等^[27]^提出基于NCC-GP150(使用TCGA设计的，覆盖150个肿瘤相关基因外显子区域的靶向基因面板)的bTMB与基于组织活检wTMB具有较好的相关性。TMB在不同肿瘤亚型中的表达水平各不相同，由于肿瘤异质性，即使在同一肿瘤类型中，TMB的表达也存在显著差异。也有研究表明，在NSCLC中，TMB与吸烟呈负相关，性别与TMB的无明显相关性，伴随驱动突变(如*EGFR*、*ALK*和*ROS1*)的患者的TMB通常较低，而部分基因(如*BRCA1*、*TP53*和*POLE*)的突变会引起TMB显著升高^[22, 28-32]^。

## 3 生物信息学管道对TMB值的影响

到目前为止，对于哪些突变应该包括在TMB计算中还没有达成共识^[33]^。2020年的一项回顾性研究表明，在计算过程中缺乏胚系突变的剔除的会导致TMB偏高^[34]^。2019年，Benayed指出有形成新抗原潜能的染色体重排可能无法被靶向基因面板检测，从而导致PTMB偏低^[35, 36]^。CheckMate 026临床试验对312例接受一线Nivolumab治疗或标准化疗的NSCLC患者进行了回顾性分析，结果表明，与仅包含错义突变的测序方式相比，包含同义突变、插入突变、移码和无义突变的测序会引起TMB水平升高，尤其是与肺腺癌一样与同义突变和错义突变有密切相关的肿瘤类型^[37]^。Buchhalter等^[21]^也建议在TMB的计算中包括所有的同义和错义突变，以提高计算的精确度。Foundation One CDx(一种基于定性的下一代测序的体外诊断试验)是通过人群数据库过滤确定肿瘤细胞内的体细胞突变，WES和MSK-IMPACT是通过患者白细胞对照过滤肿瘤样本中检测到的遗传性突变，以确保用于计算TMB的突变均为体细胞突变，Foundation One CDx则是通过SGZ(somatic-germline-zygosity)算法确定肿瘤细胞内的体细胞突变^[38]^。目前公开的人群数据库均以欧美人群为主，虽有人曾提出使用足够全面的数据库可以减轻种系有关的差异，有研究^[39]^提出在确定体细胞突变时，使用数据库进行种系突变筛选(而不是配对正常样本)会增加TMB与种族相关的异质性。在临床中，准确报告分析管道的具体参数(例如，包括的突变类型、用于过滤及识别突变的数据库)，有助于对来自不同生物学分析管道的TMB数据进行校准。2020年，德国病理学家协会(Quality in Pathology, QuIP)使用15个测试中心的6个面板，评估包含肺腺癌的三种肿瘤类型的20个样本的TMB值，提出在面板的生物信息管道中添加附加的过滤器，可以进一步改进上述提到的种系突变所引起的差异^[40]^。2020年Yao等^[41]^建议依赖于频繁更新的数据库的过滤器，也会进一步恶化计算的不一致性、再现性和稳定性。为了促进pTMB的校准及pTMB间的可比性，Friends建议，参考标准生成的校准曲线可以应用于面板的标准化和面板间的比较，*Spearman*比*Pearson*方法更适合于psTMB与WESTMB相关性的评估，线性回归模型适用于从pTMB到wesTMB的转换，TMB报告应协调为：突变/megabase(mut/Mb)^[19]^。2020年，Yao等^[41]^为了解决TMB测量的分析中不一致性以及TMB分类缺乏确定的阈值描述了一种新的方法来解决这一问题：使用统计模型对TMB进行估计和分类(Estimation and Classification of TMB, ecTMB)，并使用贝叶斯框架(Bayesian framework)对TMB进行预测以系统地纠正面板设计产生的偏差。作者提出，ecTMB比标准方法能更准确地预测背景突变。与目前的计数方法相比，ecTMB的优点还包括：①通过对面板设计偏差的系统修正，能够提高TMB值的一致性; ②ecTMB包含了同义突变; ③esTMB不是基于单个计数值来预测TMB，而是通过将每个基因视为一个独立的负二项过程来预测TMB。

## 4 DNA文库浓度、取样部位、样本保存方式和样本保存期对TMB值的影响

2020年，Valsamo Anagnostou通过对3, 788个wTMB数据进行分析，提出当样本中的肿瘤纯度低于50%时，TMB与肿瘤纯度之间的具有较明显的负相关性^[42]^。随后由Tae Hong等^[43]^指出，对接受免疫治疗的NSCLC患者，在肿瘤纯度低于30%的样本中，pTMB(本实验中使用的面吧包含381个目标基因的靶向面板，基因组大小为1.07 mbMb)仍可以提供可靠的预测性能，但是全外显子测序会严重低估TMB，由于该研究的生存分析主要基于PFS而不是总生存期(overall survival, OS)，因此，还需要通过大规模前瞻性试验加以验证靶向面板在低纯度样本中的临床价值。Quy等^[44]^对199例实体瘤患者的数据进行回顾性分析发现：①DNA文库的浓度 < 5 nmol/L时，TMB评分显著升高(*P* < 0.01); ②当DNA文库浓度均 < 5 nmol/L时，TMB与样本储存期呈正相关(*r*=0.39, *P* < 0.001, *n*=195)，如果文库DNA浓度≥5 nmol/L，TMB与样本保存时间的统计学相关性消失; ③FFPE样本与高TMB得分显著相关; ④经过放射治疗部位样本的TMB评分明显高于非辐射野的样本; ⑤TMB与化疗方案数量无相关性。理论上，在50倍的覆盖率下，95%的SNV和具有15%或更高变异等位基因频率的短插入或缺失突变可以被一致地检测到^[45]^。然而，由于非肿瘤细胞的污染、肿瘤的异质性和FFPE诱导的脱氨伪影的影响，在临床中，我们需要进行更深入的测序以保持较高灵敏度。

## 5 结语与展望

免疫治疗是肿瘤治疗的重要转折点，为肿瘤患者带来了福音。然而，并非所有患者均能从中获益，故亟需探索有效预测免疫治疗效果的生物标志物。尽管TMB已作为NSCLC患者接受免疫治疗的预测性生物标志物，但其仍存在许多局限性。如组织标本的质量和数量、肿瘤组织异质性、不同的检测平台、相对较长的检测时间、昂贵的价格等均限制了TMB的应用^[45]^。用于TMB评估的分析前因素的协调和标准化、根据肿瘤类型选择合适大小的靶向面板，对于TMB作为免疫治疗疗效的生物标志物的可靠性至关重要。不同的测序方式及生物信息学算法对TMB的评估和报告也存在着较大的影响，由于它们在不同的基因面板平台上存在很大差异，因此，需要标准化实验室的测序方式及生物信息学分析方法。TMB存在瘤内异质性，因此，单样本的TMB评估在临床工作中具有一定的局限性。虽液体活检可能不容易受到与瘤内异质性的影响^[44]^，但bTMB预测OS的能力仍有待进一步探索，需要更多的支持证据以提高bTMB临床价值。由于部分驱动基因的突变会影响肿瘤免疫原性和随后免疫调节药物的潜在疗效，并且与TMB具有明显相关性，因此在临床中，这些驱动基因与TMB一起评估是有必要的。目前对用预测免疫治疗疗效的阈值仍存在争议。在未来，尚需要更多的实验证据证实TMB对于NSCLC患者精确治疗的临床价值。
